# ChlamyCyc: an integrative systems biology database and web-portal for *Chlamydomonas reinhardtii*

**DOI:** 10.1186/1471-2164-10-209

**Published:** 2009-05-04

**Authors:** Patrick May, Jan-Ole Christian, Stefan Kempa, Dirk Walther

**Affiliations:** 1 Max-Planck-Institute of Molecular Plant Physiology, Potsdam, Germany; 2 University Potsdam, Potsdam, Germany

## Abstract

**Background:**

The unicellular green alga *Chlamydomonas reinhardtii *is an important eukaryotic model organism for the study of photosynthesis and plant growth. In the era of modern high-throughput technologies there is an imperative need to integrate large-scale data sets from high-throughput experimental techniques using computational methods and database resources to provide comprehensive information about the molecular and cellular organization of a single organism.

**Results:**

In the framework of the German Systems Biology initiative GoFORSYS, a pathway database and web-portal for Chlamydomonas (ChlamyCyc) was established, which currently features about 250 metabolic pathways with associated genes, enzymes, and compound information. ChlamyCyc was assembled using an integrative approach combining the recently published genome sequence, bioinformatics methods, and experimental data from metabolomics and proteomics experiments. We analyzed and integrated a combination of primary and secondary database resources, such as existing genome annotations from JGI, EST collections, orthology information, and MapMan classification.

**Conclusion:**

ChlamyCyc provides a curated and integrated systems biology repository that will enable and assist in systematic studies of fundamental cellular processes in Chlamydomonas. The ChlamyCyc database and web-portal is freely available under .

## Background

The unicellular green alga *Chlamydomonas reinhardtii *(for brevity, in the following referred to as Chlamydomonas) is an important eukaryotic model organism for the study of photosynthesis and chloroplast development in higher plants as well as flagella development and other cellular processes, and has recently attracted substantial interest in the context of bio-fuel and hydrogen production [1,2]. Because of its unique evolutionary position – it diverged from land-plants over a billion years ago – the genome and its gene catalogue have received much attention, especially since the recent publication of the draft genome [2]. The genome of Chlamydomonas currently (version 3.1) contains about 14,500 protein-coding genes. Additionally, the mitochondrial and plastid genomes have been fully sequenced.

Although the Chlamydomonas genome is far from being completely annotated, e.g., there are more than 150,000 alternative gene models of unclear validity available in addition to the currently annotated genes, there is a fast growing need for a better understanding of the functional aspects of Chlamydomonas. Especially in the context of metabolic network analysis, missing enzymes have to be identified, so that a fully functional network can be obtained. Such demands can best be met by an integrated Systems Biology approach, which typically includes several 'Omics' technologies combined with bioinformatics and modelling methods.

Biochemical pathway maps composed of genes, proteins, and metabolites are powerful reference models for the compilation and presentation of information derived from genomic datasets [3]. Currently, several Chlamydomonas-related web resources are available including the JGI genome browser [4], the website of the Chlamydomonas consortium [2], a database for small RNAs [5] and the new, jointly developed ChlamyBase portal [6]. But none of these Chlamydomonas-related databases or web resources listed above is capable of visualizing functional genomics data (e.g. expression data obtained by microarray analysis or proteomics) within the context of Chlamydomonas-specific biological pathways and reactions. Chlamydomonas metabolic pathway information, albeit incomplete, is currently only available from the KEGG [7] database. Tools such as PathExpress [8] and KEGG-spider [9] provide the possibility to visualize gene expression data in the context of KEGG-based pathways, sub-pathways, and metabolites. Alternatively, MapMan is a visualization platform that has been developed for the display of metabolite and transcript data onto metabolic pathways of Arabidopsis and other plant genomes [10-14] and thus features a special emphasis on plant-specific pathways.

In the post-genomic era of modern high-throughput technologies, sophisticated computational biology tools are essential to integrate the increasing amount of experimental data generated from experimental systems biology studies such as genomics, transcriptomics, proteomics, and metabolomics, for a comprehensive representation of cellular processes on all levels of molecular organization. The Pathway Tools software [15] together with the MetaCyc database [16] is a well-established method to annotate and curate high-throughput biological data in the context of metabolic pathways, gene regulation, and genomic sequences. It allows the automated generation of so-called Pathway/Genome databases (PGDBs) through functional assignment of genes and manual curation of pathways using a graphical user interface. MetaCyc consists of pathways, reactions, enzymes and metabolites together with literature information from more than 600 species, ranging from microbes to plants and human [17]. To date, several PGDBs have been created for plants species, e.g., AraCyc (*Arabidopsis thaliana*) [18], RiceCyc (Rice) [19], MedicCyc (*Medicago trunculata*) [20], or the newly established PlantCyc database [21], a comprehensive plant biochemical pathway database, but up to now no PGDB for algae or related species has been developed.

ChlamyCyc is a model-organism specific, web-accessible pathway/genome database and web-portal [22] that was developed as part of the German Systems Biology research initiative GoFORSYS (Golm FORschungseinheit SYStembiologie) [23], a systems biology approach towards the study of photosynthesis and its regulation in response to selected environmental factors in the model algal system Chlamydomonas. ChlamyCyc serves as the central data repository and data analysis and visualization platform of cellular processes and molecular responses in Chlamydomonas within the GoFORSYS project. The integration with genome databases such as JGI [24], PlantGDB [25] and Genbank, as well as cross-links to secondary databases and annotation tools like PlntTFDB [26], ProMEX [27], Quantprime [28], MapMan [10] further increases the utility of the ChlamyCyc web-portal.

## Implementation

### Data preparation

Genome, transcript, and protein sequences and corresponding annotation files for the Chlamydomonas frozen gene catalog v.3.1 (September 2007) were downloaded from the Joint Genome Institute of the U.S. Department of Energy (JGI) [29]. Plastid and mitochondrial sequences were obtained from NCBI, and Chlamydomonas EST, EST assembly, GSS, STS, and HGT sequences from PlantGDB[30], which mirrors NCBI dbEST [31], dbGSS [32], dbSTS [33], and HGTS [34] databases. Chlamydomonas tRNA, sRNA, snRNA, and microRNA sequences were downloaded from PlantGDB, cresi-RNA database [5], and MirBase [35].

All EST and EST assembly consensus sequences were mapped onto the draft genome of Chlamydomonas assembly v3.1 by GMAP [36] using a method similar to the one described in [37]. For the definition of a valid genome mapping, we used the following criteria: minimum alignment identity and the minimum coverage of the EST sequence of at least 80%. RNA sequences were aligned onto the genome using the RazerS [38,39] software, a tool for fast and accurate mapping of short sequence read against genome sequences. Table 1 shows all available Chlamydomonas data that were used to build the ChlamyCyc database and genome browser.

**Table 1:** Chlamydomonas sequence data collected for the ChlamyCyc web-portal

**Name**	**Type**	**Source**	**Mapping**
	**Genomic sequence data**		
Chlre3_1_genome_scaffolds	DNA genomic scaffolds (all^1^)	JGI^2^	-
JGI 4.0 genome_scaffolds	DNA genomic scaffolds	JGI^3^	-
Chlamy chloroplast	DNA complete genome sequence (. NC_005353)	Genbank	-
Chlamy mitochondrion	DNA complete genome sequence (. NC_001638)	Genbank	-
Chlamy GSS	DNA (15574 GSS sequences from Genbank^4^)	PlantGDB^5^	razerS^12^
Chlamy STS	DNA (8 STS sequences from Genbank^7^)	PlantGDB^5^	razerS^12^
Chlamy HTG	DNA (2 HTG sequences from Genbank^8^)	PlantGDB^5^	razerS^12^
			
	**Transcript sequence data**		
Chlre3_1.GeneCatalog_2007_09_13.transcripts	mRNA transcripts (frozen gene catalog 3.1)	JGI^2^	GFF (from JGI)
Chlamy Chlre3.1M dna	mRNA transcripts (Science paper)	JGI^2^	GFF (from JGI)
Chlre3_1_allESTs.fasta	mRNA transcripts	JGI^2^	gmap^6^
Chlre3_1_ESTcluster.cr.171	mRNA transcripts	JGI^2^	gmap^6^
Chlre3_1_ESTcluster.cr.210	mRNA transcripts	JGI^2^	gmap^6^
Chlamy ESTs	mRNA (202044 EST sequences from Genbank)	PlantGDB^4^	gmap^6^
Chlamy ESTcontigs	mRNA (50380 EST assembly sequences)	PlantGDB^4^	gmap^6^
	*RNA data*		
microRNAs	RNA (microRNAs)	MirBase^10^	GFF (from JGI)
Cresi-RNAdb	RNA (small RNAs)	cresi-RNA^11^	razerS^12^
Chlamydomonas_reinhardtii.scRNA.PLN	RNA (1 scRNA from Genbank)	Genbank	razerS^12^
Chlamydomonas_reinhardtii.snRNA.PLN	RNA (5 snRNAs from Genbank)	Genbank	razerS^12^
Chlamydomonas_reinhardtii.tRNA.PLN	RNA (7 tRNA sequences from Genbank)	Genbank	razerS^12^
Chlamydomonas_reinhardtii.RNA.PLN	RNA (4182 RNA sequences from PLN nucleotides)	Genbank	razerS^12^
			
	**Protein sequence data**		
Chlre3_1.GeneCatalog_2007_09_13.proteins	Protein (Frozen gene catalog 3.1)	JGI^2^	GFF (from JGI)
Chlamy Chlre3.1M.pep	Protein (Science Paper)	JGI^2^	GFF (from JGI)
allChlre3.proteins	Proteins (alternative gene models)	JGI^2^	GFF (from JGI)
Chlamy cp aa	Proteins (chloroplast)	Genbank	Genbank
Chlamy mt aa	Proteins (mitochondrion)	Genbank	Genbank
Chlamy peptides	Proteins (experimental identified peptides)	JGI^3^	JGI

^1^Assembly v.3.1 scaffolds and scaffolds excluded from v.3.1 assembly file based on blast hits or manual examination

^2^

^3^Personal communication

^4^

^5^

^6^

^7^

^8^

^9^

^10^

^11^

^12^

### Annotation process

MapMan is an ontology developed to capture the functional capabilities of higher plants [10-12]. It has been recently adapted to the Chlamydomonas genome [40]. MapMan annotation was generated by assigning current Chlamydomonas proteins to MapMan categories using Blast[41] searches (NCBI Blast version 2.2.16) against plant proteins, which had previously been classified using the MapMan classification system. All Blast-derived hits with bit-scores of 50 or less were excluded from further analysis. Furthermore, all sequences were scanned for known motifs and/or families using Interproscan [42]. The results were combined with manual annotation to provide a draft classification of the Chlamydomonas encoded proteins from all three available genomes.

**Figure 1:**
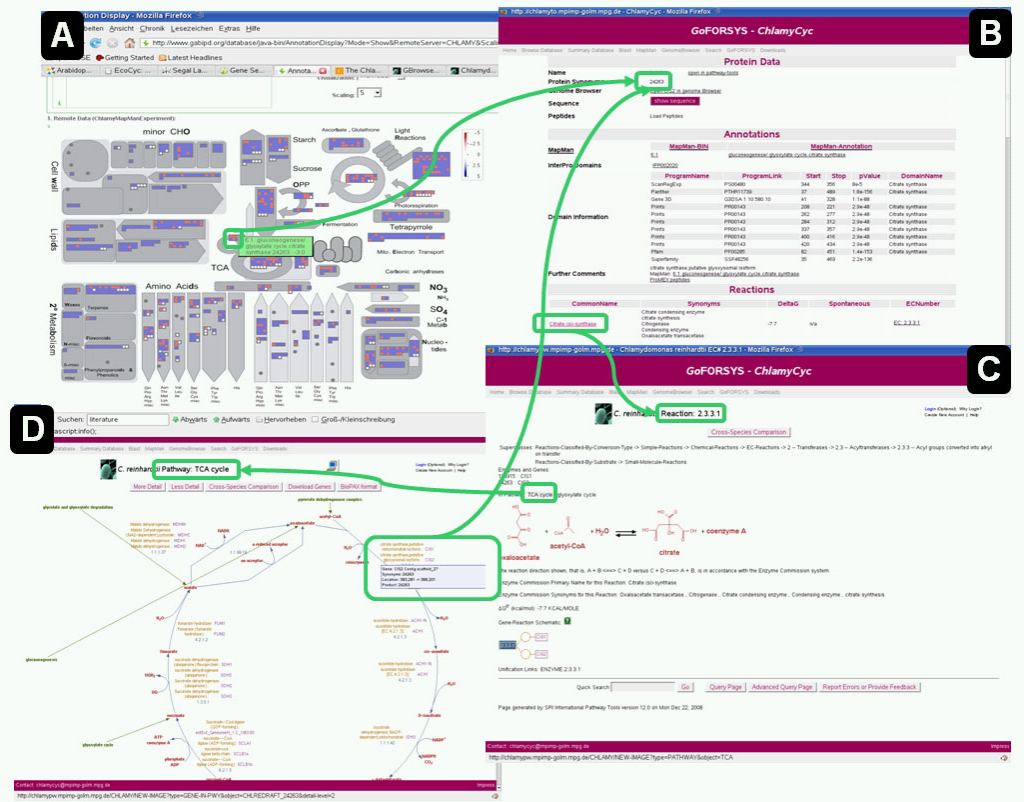
**Chlamydomonas central metabolism pathways and processes**. (A) The Chlamydomonas central metabolism is visualized using the MapManWeb [58] visualization tool. Squares represent Chlamydomonas proteins that have been assigned into the various MapMan metabolic pathways depicted on the diagram. These are colored red if matching peptides have been found by proteomics and blue otherwise. All proteins are linked to their ChlamyCyc gene pages (1). Metabolites that have been identified experimentally are represented by white boxes. (B) Gene page for the citrate synthase gene CYS1 containing information and links to annotation data, orthologs, and metabolic reactions (2). (C) Enzymatic reaction page for reaction EC2.3.1.1 with links to pathways containing this reaction (3). (D) TCA cycle. The proteins are linked back to their corresponding protein and gene pages (4). Links between web pages are shown in green.

We further annotated all JGI v3.1 proteins by their peptide support derived from proteomics analysis [40]. A total of 4,202 experimentally validated peptides were identified to map uniquely to 1,069 proteins [40] (see Figure 1A). Within ChlamyCyc, we link these proteins to the plant proteomics mass spectral reference library ProMEX [43], where the mass spectra can visualized and further analyzed.

To evaluate the completeness of the Chlamydomonas metabolic reconstruction, we compared the postulated metabolic compounds in ChlamyCyc with compounds that have been identified in metabolic profiling experiments using GC-TOF-MS [44], GCxGC-MS [40], and GCxGC-TOF-MS [45]. From the 155 metabolites reported in the two studies, 149 were part of the BioCyc Open Chemical Database (BOCD) and, therefore, part of the MetaCyc database [16] (see Additional File 1). These metabolites were inserted manually into the ChlamyCyc database together with their corresponding literature annotations. The six missing metabolites were submitted to the BOCD for inclusion in upcoming releases of the MetaCyc database. In Figure 1A, the identified metabolites are highlighted in the context of their metabolic pathways and processes.

Functional annotation is normally done by transferring functional information across organisms using comparative analysis. Therefore, inferring the correct orthology and paralogy relationships is a crucial step in the annotation process. For the establishment of equivalences among genes in different genomes, homology alone is often not sufficient. We used the Inparanoid [46] software and the OrthoMCL-DB database to obtain evolutionary relationships between Chlamydomonas and other species. With Inparanoid, we found 6,219 pairwise orthology groups for 10,406 Chlamydomonas genes against 24 different organisms. Downloading the OrthoMCL-DB[47,48], we obtained 6,130 orthology groups with at least one Chlamydomonas gene. In total, we found orthologs to 10,398 Chlamydomonas genes in 86 species. The KEGG Orthology (KO) system is a classification system of orthologous genes, including orthologous relationships of paralogous gene groups [7,49]. Currently, there are 2,540 Chlamydomonas genes annotated into 1,866 KO groups. In total, for 12,489 Chlamydomonas genes an orthology relationship could be obtained (see Additional File 2). All orthology and paralogy relationships are provided in the Additional Files 3, 4, and 5.

From the plant-specific transcription factor database PlnTFDB [26], we obtained annotations for 211 Chlamydomonas transcription factors. These proteins were linked back to the PlnTFDB. A list of all annotated transcription factors can be found in Additional File 6.

### Construction

The ChlamyCyc metabolic pathway database was constructed using MapMan annotations, cross-species orthology assignments, as well as available annotations from KEGG [7] and JGI [24]. Chlamydomonas genomic, transcript, and protein sequences were downloaded from JGI. Due to the currently still incomplete status of the Chlamydomonas genome sequencing, not all genomic scaffolds have been associated with chromosomes. Therefore, transcripts were associated with their assigned scaffolds and, if possible, the 17 annotated Chromosomes. The KEGG, JGI, MapMan, and our comparative annotation of EC (Enzyme Commission) numbers and GO terms were formatted into a PathoLogic-specific set according to the documentation for Pathway Tools [50] and used for the first ChlamyCyc database construction. Enzymes labeled as 'putative' or 'similar to' were also included in the dataset. The initial ChlamyCyc database was generated using the PathoLogic Pathway Prediction module of Pathway Tools version 11.5. The initial Chlamydomonas pathways were inferred using MetaCyc 11.5 as a reference database of metabolic pathways using AraCyc and YeastCyc PGDBs as co-reference databases. Afterwards, the pathways, reactions, compounds were curated manually. The current version of ChlamyCyc uses the upgraded Pathway Tools version 12.5.

## Results

### ChlamyCyc statistics

**Figure 2:**
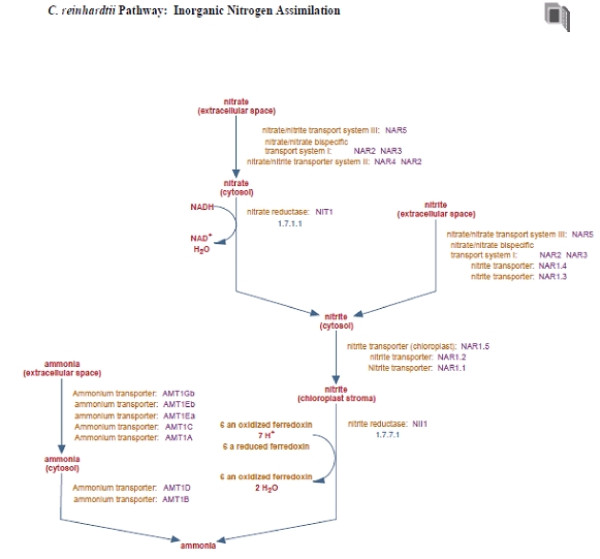
**Inorganic Nitrogen Assimilation pathway**. Displayed in the Pathway Tools pathway browser.

The initial and automated construction of ChlamyCyc (ChlamyCyc version 1.0) with the PathoLogic software contained 2,794 enzymes for which functional annotation was known, and another 272 gene products identified as 'probable enzymes'. The 'probable enzymes' consisted of generic annotations such as 'Methyltransferases' and the precise functions of these enzymes are still unknown. In total, the initial ChlamyCyc version 1.01 database contained 243 pathways (see Table 2) comprising 1,346 enzymatic reactions. After manual reconstruction and computational consistency checks (see [40,51] for details), the final curated version of ChlamyCyc (1.0.1) covers 253 pathways, 1,419 enzymatic reactions, 2,851 enzymes, and 1,146 compounds (see Table 2). 928 literature citations were added manually or were included from the gene annotation at the JGI genome browser [29]. Figure 2 shows the Chlamydomonas specific 'Inorganic Nitrogen Assimilation' pathway as defined in literature [52,53]. All ChlamyCyc data sets are downloadable in Pathway Tools flat files or SBML format from the ChlamyCyc web-page [54].

**Table 2:** Overview of data content in ChlamyCyc (version 1.0.1)

**Data type**	**Number**
Pathways	243
Enzymatic Reactions	1,419
Transport Reactions	15
Polypeptides	14,653
Protein Complexes	4
Enzymes	2,851
Transporters	58
Compounds	1,146
microRNAs	36
tRNAs	32
rRNAs	24
Regulations	14
Literature citations	928

### MapMan annotation

In total, we could annotate 5,359 nuclear-encoded proteins onto non-trivial MapMan classification bins [12] covering more than one third of the currently annotated proteins in Chlamydomonas (see 1A). The 67 annotated proteins known to be organelle-encoded were classified manually based on their gene name and available literature information. The functional MapMan classification of Chlamydomonas proteins was made available as a webservice using the Perl BioMoby API (API [55] on a standard server running SUSE Linux). For the new ChlamyCyc web-portal, we implemented the Chlamydomonas MapMan classification hierarchy as a searchable web interface [56] linking the annotated proteins directly to the ChlamyCyc pathway database or gene-specific pages (see below). The Chlamydomonas MapMan classification can further be visualized using the MapManWeb [40,56-58] tool that is linked directly from the ChlamyCyc web-portal (see also Figure 2A). The MapMan stand-alone software tool including visualization of Chlamydomonas experiments is available from [59]. The MapMan annotation for Chlamydomonas can also be found in Additional File 7.

### Gene-specific pages

The ChlamyCyc gene pages integrate genomic, proteomic as well as functional annotation data. Genomic data comprise genomic mapping information, sequences and available validated or predicted primer information using information directly obtained from Quantprime [28]. Every gene can be visualized directly in its genomic context in the ChlamyCyc genome browser. Protein-related data is represented by sequence information, experimentally validated peptides [40], annotation links to Uniprot [60], the GO ontology [61], and predicted proteotypic peptides for quantitative proteomics using PeptideSieve [62]. For every protein, the ChlamyCyc reactions, the MapMan annotation, domain predictions from InterPro [63] and Pfam [64] are presented. Additionally, all orthologous and paralogous genes from KEGG KO, Inparanoid, and OrthoMCL-DB are shown and all sequences are downloadable.

### Chlamydomonas Genome Browser

**Figure 3:**
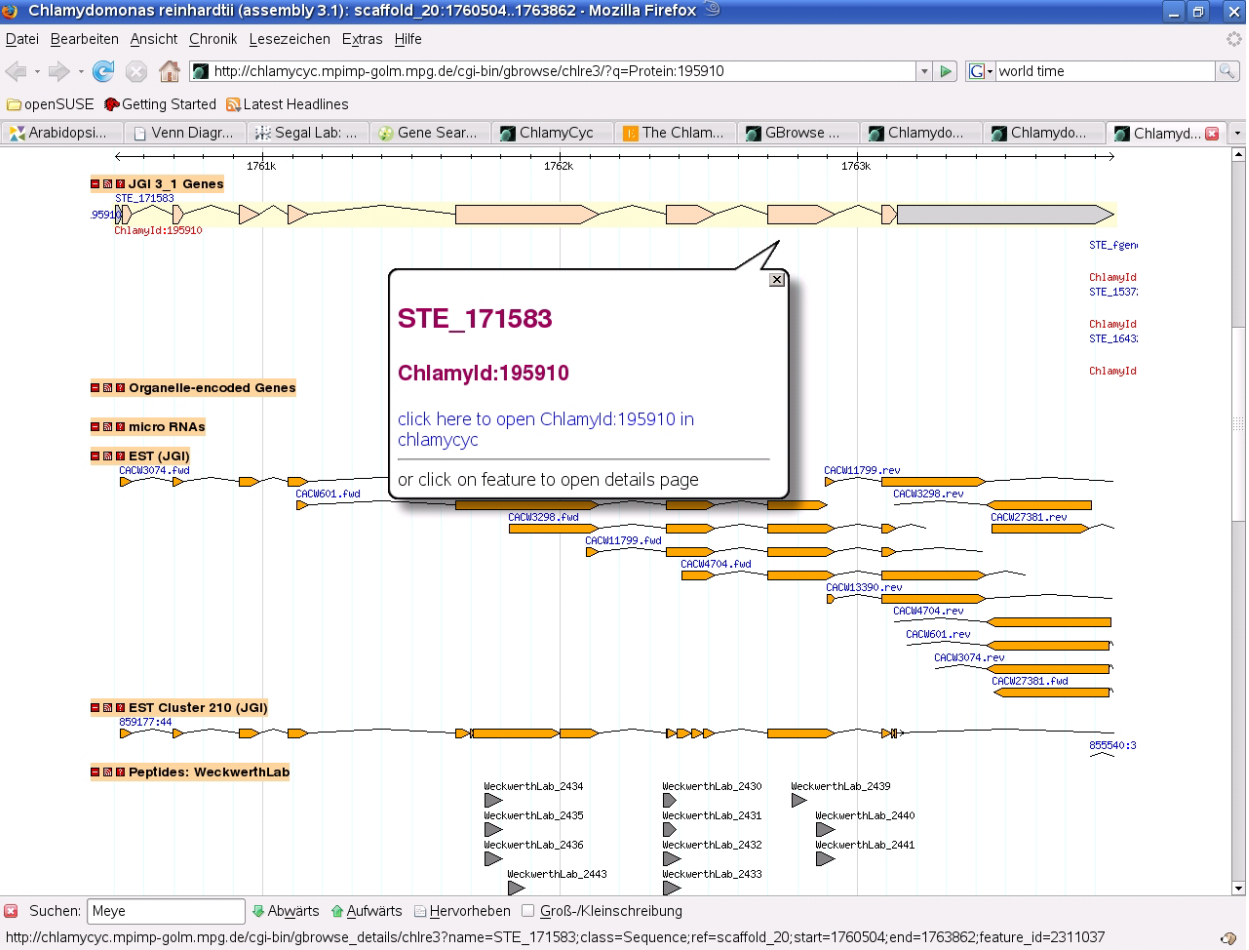
**Example of the ChlamyCyc genome browser functionality**. Shown is the Chlamydomonas gene Chlredraft_195910 in the ChlamyCyc genome browser together with supporting EST and peptide data (WeckwerthLab) obtained from proteomics experiments [40]. Every gene is directly linked back to the ChlamyCyc gene pages.

We implemented a Chlamydomonas specific genome browser based on the GBrowse software package [65]. For its implementation for the Chlamydomonas genome, we used the genomic scaffold and genome information of JGI version 3.1 as available from the JGI website [29] as well as the Chlamydomonas plastid and mitochondrial genome as available from NCBI Genome [66]. We added tracks for annotated transcripts, proteins, and RNAs for the three available genome sequences (see Figure 3). Additionally, we added tracks for the proteomics data (as kindly provided from JGI) (see Table 1) and our in-house experimental studies [40]. The Gbrowse window can be used to display individual and user-defined combination tracks for Chlamydomonas data. Table 1 provides an overview of all Chlamydomonas sequence data that is available through the ChlamyCyc genome browser [4].

### Chlamydomonas Blast search

A web version of the standard Blast software [7] customized for the Chlamydomonas annotation was implemented as part of the ChlamyCyc web-portal. Sequences in Fasta format can be searched against all available Chlamydomonas genomic, transcript, RNA, and protein sequences databases. A list of all available sequence sets together with a short description and corresponding data sources are given in Table 1. If a matching hit of the query sequence to an annotated protein-coding gene is found, the Blast results are linked directly to the ChlamyCyc gene pages, and in case of matching hits against alternative gene models that are not annotated in ChlamyCyc, to the corresponding gene-specific website at JGI [29].

### Visualization Tools

Various functional genomics data from gene expression, protein expression, and metabolic profiling experiments can be visualized in the context of the reconstructed metabolic network of Chlamydomonas using either the Pathway Tools Omics Viewer [3] or the MapMan [58] tools as described above. Both visualization tools enable the visualization of the user's own data. In addition, the Chlamydomonas genome browser allows to upload customized user tracks, e.g. from gene expression studies.

## Discussion

ChlamyCyc, a curated and integrated Pathway/Genome database (PGDB) and web-portal for Chlamydomonas, was developed to enable and assist in further studies of metabolism and functional genomics in Chlamydomonas. The goals of this project were: (i) to use metabolic network reconstruction for predicting the metabolic composition of Chlamydomonas; (ii) to provide a platform for visualization of the integrated functional genomics datasets. Furthermore, long-term goals are: (iii) to contribute towards the functional annotation of as yet uncharacterized genes and gene products via comparison with other sequenced plant genomes and detected metabolites; and (iv) to provide curated resource for the study of photosynthesis, growth, and energy production in Chlamydomonas.

The Pathway Tools [15] software gives us the possibility to build a model organism database for Chlamydomonas including species-specific pathway and literature data. Additionally, we adapted the MapMan [12,40] ontology to annotate the Chlamydomonas gene repertoire and to visualize data from various 'Omics' techniques. MapMan has been chosen because of its special emphasis on photosynthesis-related and other plant-specific pathways. Both methods will enable us in the future to incorporate new information concerning the Chlamydomonas metabolism as well as to define Chlamydomonas-specific gene classifications from a plant-specific context.

ChlamyCyc results from a genome-scale metabolic pathway reconstruction to generate a pathway database for Chlamydomonas. ChlamyCyc was assembled based on the recently published genome sequence [2] and MapMan annotations of Chlamydomonas genes using the Pathway Tools software within the BioCyc family of databases. The predicted pathways were verified using orthology information from various other species and manual curation. We analyzed and integrated a combination of database resources, such as existing genome annotations from the genome project at JGI, databases like PlnTFDB [26] and ProMEX [27], EST collections, and protein domain scanning as well as literature information.

Chlamydomonas genomic sequencing and advances in mass spectrometry have enabled large-scale profiling of proteins [40,67,68]. In several metabolomics studies, a variety of metabolites could be identified [40,44]. In addition to pathway information, comprehensive gene-based annotation has been gathered and made available for all currently identified genes in the Chlamydomonas genome via custom gene report pages. ChlamyCyc is cross-linked to currently 13 other Pathway Tools instances of other organisms that are of greatest relevance for the study of Chlamydomonas including 8 other plant species, the new PlantCyc database for crop plants as well as *E. coli*, Yeast, Synechocystis, and Human allowing comprehensive cross-genome metabolic pathway analyses [69]. The utilization of a common BioCyc database format provides a consistent platform for the comparison of reconstructed pathways between Chlamydomonas and other available PGDBs. This is easily possible by using the Pathway Tools comparative module. Direct comparisons between Chlamydomonas and other plant or fungi may also reveal current gaps in the knowledge of Chlamydomonas metabolism.

Since the annotation of the Chlamydomonas genome is an ongoing project, and by far not all gene models are confirmed or validated using experimental data, we decided to integrate all available gene models into our Chlamydomonas genome browser together with the current annotation as available from JGI. This gives us (and the user) the possibility to visualize alternative gene models together with tracks showing their peptide support as measured in proteomics studies. Ultimately, the correct gene structure for all Chlamydomonas genes will await the completion of the JGI genome sequencing and assembly. Until such experimental confirmation for all gene models exists, the comparison of different predictions may offer a good starting point for judging the reliability of the annotated and alternative gene structures. We used the Generic Genome Browser (GBrowse) [6] platform to establish a comparative view of the different genome annotations as available from JGI together with annotations for the plastid and mitochondrial genomes and additionally available experimentally validated peptide data.

As the Chlamydomonas genome sequencing project moves toward the completion of version 4.0, ChlamyCyc will be updated accordingly. Continued curation will be necessary to address new gene annotations and new metabolic pathways related to Chlamydomonas metabolism, which are becoming available during on-going sequencing and annotation of the Chlamydomonas genome. One next step will be the direct linking of ChlamyCyc reactions with experimentally derived metabolomics data in the currently updated version of the Golm Metabolome Database (GMD) [70,71]. Future efforts will focus on the inclusion of subcellular localizations for specific known enzymatic isoforms and metabolites. Continuing gene expression and metabolic profiling experiments and proteomics studies are expected to provide additional information concerning cellular processes in Chlamydomonas and will be added as they are becoming available. It is anticipated that the ChlamyCyc resource will serve as a repository and common reference system for our current and future understanding of Chlamydomonas cellular processes, provide a fundamental tool for the visualization of functional genomics datasets, become integrated into larger databases (MetaCyc, ChlamyBase, JGI, etc.), facilitate comparative studies of pathways across species, and enable the prediction and annotation of Chlamydomonas specific cellular processes.

## Conclusion

ChlamyCyc provides a curated and integrated systems biology repository that will enable and assist in systematic studies of fundamental cellular processes in Chlamydomonas. The ChlamyCyc database and web-portal is freely available under .

## Availability and requirements

Project name: ChlamyCyc

Project home page: [22]

Other requirements: None

License: None required

Any restrictions to use by non-academicians: None

## Abbreviations

BOCD: BioCyc Open Chemical Database; EST: expressed sequence tags; GMD: Golm Metabolome Database; GSS: genome survey sequences; HTG: high-throughput genomic sequences; JGI: Department of Energy Joint Genome Institute [24]; KEGG: Kyoto Encyclopedia of Genes and Genomes; PGDB-Pathway/Genome database; SBML: Systems Biology Markup Language; STS: sequenced tagged sites.

## Authors' contributions

PM and DW initiated and coordinated the project. PM collected all the data, did the annotation work, set up the ChlamyCyc within Pathway Tools, curated the databases and annotations, and wrote the manuscript. JOC was responsible for setting up the web server, implementing the web portal and genome browser, and incorporating new data. SK contributed to the analysis of the metabolites and the pathway curation. All authors read and approved the final manuscript.

## Supplementary Material

Additional file 1 **Chlamydomonas metabolites identified by GCxGC-MS**. Excel file containing the metabolites found by mass spectronomy[40] together with their ChlamyCyc ID and the description if they were manually included ('added') into ChlamyCyc or part of the initial draft network ('ok'). Click here for file

Additional file 2 **Chlamydomonas orthology relationships**. Venn diagram for the Chlamydomonas orthology relationships obtained from Inparanoid, OrthoMCL-DB and KEGG-KO (for details main manuscript). The yellow boxes show the total number of ortholog groups per method. Click here for file

Additional file 3 **Chlamydomonas orthology relationships using Inparanoid**. Tab-separated CSV-file containing the Chlamydomonas protein ids and annotated orthologs from Inparanoid [46]. Click here for file

Additional file 4 **Chlamydomonas orthology relationships from OrthoMCL-DB**. Tab-separated CSV-file containing the Chlamydomonas protein ids and annotated orthologs and OrthoMCL-DB [48]. Click here for file

Additional file 5 **Chlamydomonas orthology relationships from KEGG-KO**. Tab-separated CSV-file containing the Chlamydomonas protein ids and annotated orthologs from KEGG Orthology Database [7]. Click here for file

Additional file 6 **Chlamydomonas transcription factors from PlantTFDB**. Excel file containing the Chlamydomonas proteins annotated as transcription factors from [26]. Click here for file

Additional file 7 **Chlamydomonas MapMan annotation**. Chlamy_mapman.xls: Excel file containing the Chlamydomonas MapMan annotation. Click here for file
