# Supplementary figures and images for: ChlamyCyc: an integrative systems biology database and web-portal for Chlamydomonas reinhardtii
Source: BMC Genomics. 2009 May 4;10:209. doi: 10.1186/1471-2164-10-209 (PMC2688524; doi:10.1186/1471-2164-10-209)

## Slide 1
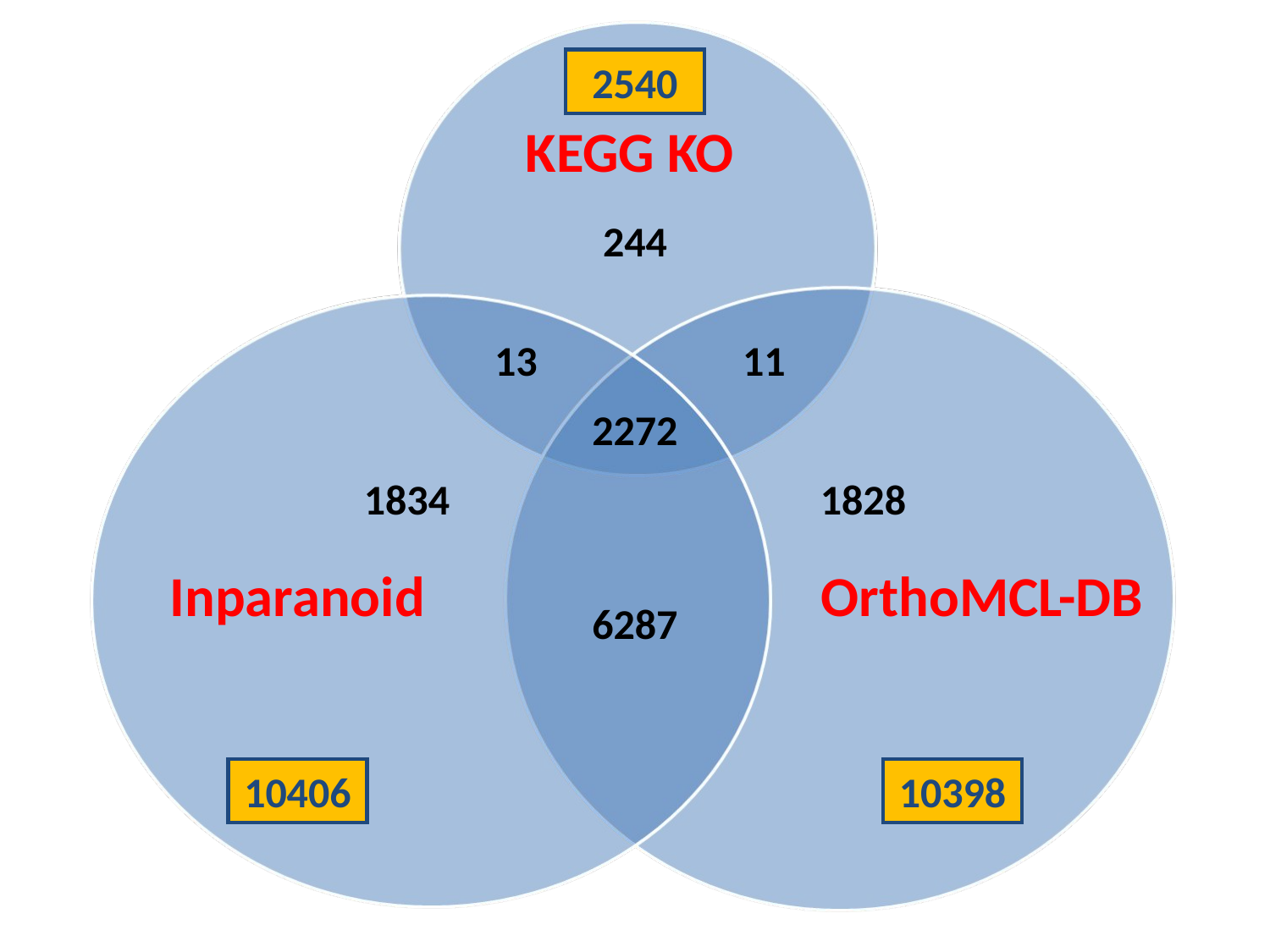

2540
KEGG KO
244
13
11
2272
1834
1828
Inparanoid
OrthoMCL-DB
6287
10406
10398

Supplement: Additional file 7 — Chlamydomonas MapMan annotation. Chlamy_mapman.xls: Excel file containing the Chlamydomonas MapMan annotation. [file 1471-2164-10-209-S7.ppt]
